# The cell type dependent sorting of CD9- and CD81 to extracellular vesicles can be exploited to convey tumor sensitive cargo to target cells

**DOI:** 10.1080/10717544.2022.2162161

**Published:** 2022-12-29

**Authors:** Stefania Zuppone, Natasa Zarovni, Riccardo Vago

**Affiliations:** a Urological Research Institute, Division of Experimental Oncology, IRCCS San Raffaele Scientific Institute, Milano, Italy; b Exosomics S.p.A, Siena, Italy; c Faculty of Medicine and Surgery, Università Vita-Salute San Raffaele, Milano, Italy

**Keywords:** Extracellular vesicles, EVs, cargo sorting, tetraspanin-based fusion proteins, cathepsin B-controlled release

## Abstract

Extracellular vesicles (EVs) are lipid membrane-bound particles involved in cell-to-cell communication through a delivery of regulatory molecules essential for physiological processes. Since EVs efficiently vectorize specific cargo molecules, they have been proposed as suitable vehicles for therapeutic agents. Drug loading into EVs can be achieved by active, exogenous strategies or by genetic modifications of vesicle-producing cells. With the aim to produce EVs conveying therapeutic proteins, we genetically engineered and compared HEK293 to tumor cells. Tetraspanin-based RFP fusions were found to be more stable and preferentially sorted into EVs in HEK293. EVs isolated from genetically modified HEK293 cells media were captured by cancer cells, efficiently delivering their cargo. Cathepsin B cleavage site introduced between CD9/CD81 and RFP was recognized by tumor specific proteases allowing the release of the reporter protein. Our results indicate HEK293 cells as a preferential system for the production of EVs and pave the way to the development of nano-platforms for the efficient delivery of therapeutic proteins and prodrugs to tumor cells.

## Introduction

Over the last years, extracellular vesicles (EVs) have emerged as appealing drug nanocarriers due to several advantages linked to their natural origin compared to synthetic nanoparticles. In fact, they lack immunogenicity and cytotoxicity, resemble body’s own cells molecular composition, improve drug bioavailability by decelerating mononuclear phagocyte system-mediated clearance and ensure an innate stability in biological fluids. These features, along with the average nano-scaled size (30–150 nm for exosomes) that allows the easy penetration into almost all body districts and the crossing of biological barriers, the cargo protection and customizable surface, make these vesicles ideal vehicles for targeted drug delivery (Kooijmans et al., 2016). Exosomes originate from the inward budding of the membrane of late endosomes, that results in the formation of vesicles inside the lumen of multi-vesicular bodies (MVBs), that in turn can fuze either with lysosome for degradation or with cell plasma membrane, releasing their vesicular content in the extracellular environment. Therefore, exosome molecular composition is determined by MVB biogenesis and includes cell distinctive nucleic acids, soluble proteins including transport proteins and heat shock proteins, and membrane proteins such as tetraspanins. As EVs molecular composition is critical determinant of their function(s) in biological processes (van Niel et al., 2018), it is very important to select the proper cell type as origin of EVs to be used for a therapeutic purpose. To-date investigated approaches to incorporate drugs into EVs can be classified into active and passive loading strategies. The former employs mechanical (extrusion), chemical (incubation with or without permeabilization agents, like saponin or other detergents) or physical stimuli (sonication, electroporation, freeze and thaw cycles) to incorporate small molecules (curcumin, paclitaxel, doxorubicin), RNAs or proteins (catalase, saporin) into purified EVs (Alvarez-Erviti et al., 2011; Fuhrmann et al., 2015; Haney et al., 2015; Kim et al., 2016; Nakase et al., 2015; Sato et al., 2016). Despite some of these methods have proven to be successful, they suffer from the great limitation of affecting vesicles structural composition and achieving very low drug encapsulation efficiency. Surface decoration with targeting moieties and cargo loading of EVs can also be achieved throughout genetic engineering of donor cells. The first proof of concept of the applicability of this strategy has been given by Alvarez-Erviti et al., who used Lamp2b, a well-characterized EV membrane protein, as anchor and fusion partner for the targeting domain of interest (Alvarez-Erviti et al., 2011). This seemingly simple yet effective engineering technique was subsequently applied to incorporate different ligands, tags or reporter proteins into EVs (Hung and Leonard, 2015; Lai et al., 2014; Tian et al., 2014; Wang et al., 2017) used for *in vivo* delivery to specific target cells. We have focused on using tetraspanins as abundant structural EV proteins to sort proteins of interest into cell-produced vesicles. Recombinant fusions based on tetraspanins carrying reporter proteins, (i.e. CD63-GFP) are commercially available and can be easily purchased worldwide. However, despite the clear advantages that this loading method might achieve, including the increased homogeneity of vesicles batches, surface structure preservation and reduced time-consuming work, degradation of fusion proteins may occur instead of sorting into EVs, as recently demonstrated (Hung and Leonard, 2015). One solution that can favor the release of the active protein and its escape from the fate of transmembrane proteins after EV uptake, takes advantage of the over-expression of proteases within tumor cells and microenvironment. One of the common approaches used so far stands in the coupling of a therapeutic moiety to an inactivating peptide substrate sensitive to a specific protease, whose activity results in the release of the cytotoxic payload in the diseased district. Alternatively, therapeutic agents can be linked to carrier molecules through a protease-sensitive peptide bridge (Seymour, 1992). In such a contest, cathepsin B cysteine protease overexpressed in tumors, has been largely employed to activate prodrugs fused to carrier macromolecules throughout the recognition of a cleavage site, leading to the accumulation of the active cytotoxic compound in tumor cells.

In this report, we propose an innovative approach based on the genetic engineering of healthy cells to produce EVs conveying tumor-sensitive fusion proteins. To this purpose, we set up an experimental plan based on three main steps, aiming at the identification of the all key components of the system, including: (1) the ideal cell type to use as a donor for therapeutic EVs, by comparing healthy versus tumor cells; (2) the most suitable cargo-anchoring EV marker, considering both tetraspanins or single transmembrane proteins; (3) a cleavage site recognized by proteases specifically expressed by cancer cells. These three components ensure key requirements of a tumor delivery system: efficiency and cost effectiveness, safety and flexibility.

## Materials and methods

### Cells culture

Human bladder (5637) and cervical (HeLa) cancer cell lines and HEK293 human embryonic kidney cell line were maintained in RPMI 1640 medium (5637) or DMEM (HeLa and HEK293) supplemented with 10% FCS, 2 mM L-Glutamine and antibiotics (100 U/mL penicillin and 100 μg/mL streptomycin-sulfate).

### Preparation of Lamp2b, CD9, CD63 and CD81-based constructs

Total RNA from HEK293 cells was extracted using TRIzol LS Reagent (Invitrogen) according to the manufacturer’s recommended protocols. RNA was then retrotranscribed with High Capacity cDNA Reverse Transcription Kit (Applied Biosystems). The obtained cDNA was used as a template to amplify the sequences encoding Lamp2b (including the signal peptide), CD9, CD63 and CD81 genes by PCR, introducing the restriction sites NheI at N-terminus and SalI at C-terminus. RFP sequence was amplified from the pDsRed2-N1 plasmid purchased from Clontech and SalI and NotI restriction sites were inserted at N and C-terms respectively. Cathepsin B specific Phe-Leu dipeptide cleavage site (CS) or its nonspecific variant Gly-Ser (nonactive cleavage site, NACS), flanked by two glycine as a spacer and SalI restriction site were included in RFP forward primers (Primers are listed in Table 1). Lamp2b/CD9/CD63/CD81 fragments were ligated with RFP and the resulting products were amplified by PCR using the external primers and cloned in the pcDNA3.1(+) vector, previously digested with NheI and NotI. The complete sequences contain 5′-NheI-SP-Lamp2b/CD9/CD63/CD81-SalI-GFLG/GGSG-RFP-NotI-3′.

**Table 1.** List of primers used for the preparation of Lamp2b/CD9/CD63/CD81-RFP constructs.

PRIMER	SEQUENCE
SP-Lamp2b	
SP-Lamp2b F	5′-ataggctagcggtcgccaccatggtgtgcttccgcctct-3′
SP- Lamp2b R	5′- atccgtcgactgcagagtctgatatccagcata −3′
RFP CS/NACS	
RFP-CS F	5′-tgcagtcgactggctttcttggaatggcctcctccgagaacg-3′
RFP- NACS F	5′-tgcagtcgactggcggttctggaatggcctcctccgagaacg-3′
RFP R	5′-agtcgcggccgctacaggaacaggtggtggc-3′
CD9/CD63/CD81	
CD9 F	5′-ataggctagcgccaccatgccggtcaaaggaggca-3′
CD9 R	5′-atccgtcgactggaccatctcgcggttcctg-3′
CD63 F	5′-ataggctagcgccaccatggcggtggaaggaggaa-3′
CD63 R	5′-atccgtcgactgcatcacctcgtagccacttc-3′
CD81 F	5′-ataggctagcgccaccatgggagtggagggctgc-3′
CD81 R	5′-atccgtcgactggtacacggagctgttccgg-3′

### Stable transgene expression

5637, HEK293 or HeLa cells (2 x 10^6^) were plated in a 10 cm^2^ dishes and incubated for 24 hours. Transfection was performed by mixing 8 μg/dish of plasmid DNA with TransIT-X2 transfection reagent (Mirus) in a 1:2 ratio in serum- and antibiotics-free medium. After 16 hours the medium was replaced with fresh one. To obtain stable clones, cells were selected with G418 (800 μg*/*mL) for 3–4 weeks.

### EVs isolation

Cells (5 x 10^6^) were seeded on 150 mm dishes in DMEM supplemented with 10% EV-depleted FCS, 2 mM L-Glutamine and antibiotics. After 72 hours incubation, the cell culture media were collected and centrifuged (1500 rpm) for 25 min to remove cell debris. For EVs isolation, the obtained supernatant was filtered through 0.22 μm filter before ultracentrifugation at 150.000 g for 2 hours at 4° C (Beckman Coulter). The EV-containing pellet was resuspended in PBS. Concentration of isolated EVs was calculated as protein concentration, which was determined using a Pierce BCA protein assay kit (Thermo Scientific).

### Nanoparticle tracking analysis

Nanoparticle tracking analysis was performed on isolated vesicles by employing a NanoSight LM10-HS microscope (NanoSight Ltd., Amesbury, UK), as previously described (Zarovni et al., 2015). The NanoSight system was calibrated with polystyrene latex microbeads. Videos were analyzed with NTA software version 2.3 to determine the concentration and size of measured particles with corresponding standard error (SEM).

### Transmission electron microscopy

Freshly purified EVs from cultured media of CD9/CD63-RFP stably expressing HEK293 were absorbed on glow discharged carbon coated formvar copper grids, washed with water, contrasted with 2% uranyl acetate and air-dried. Grids were observed with a Zeiss LEO 512 transmission electron microscope. Images were acquired by a 2k x 2k bottom-mounted slow-scan Proscan camera controlled by EsivisionPro 3.2 software.

### Western blot analysis

Cells were washed twice with cold PBS, collected by scraping and centrifuged for 5 min at 1200 rpm. Cells were lysed for 30 min on ice in ice-cold buffer (150 mM NaCl, 2 mM NaF, 1 mM EDTA, 1 mM EGTA, 1 mM Na3VO4, 1 mM PMSF, 75 mU/ml aprotinin, 50 mM Tris-HCl, pH 7.5) containing 1% Triton X-100 and a proteinase inhibitors cocktail (Sigma-Aldrich). Cell lysates were centrifuged at 10.000 g at 4° C for 10 min. Proteins contained in the supernatant were quantified, resuspended in sample buffer (62.5 mM Tris-HCl (pH = 6.8), 2% SDS, 10% glycerol, 0.002% bromophenol blue, 5% 2-mercaptoethanol) and boiled at 95 °C for 5 minutes. To detect tetraspanins, samples were resuspended in non-reducing sample buffer. Proteins were separated though SDS-PAGE, transferred onto a nitrocellulose membrane, incubated with 5% nonfat powdered milk in TBS-T (5% Tween-20) for 1 hour and then with the following antibodies: cathepsin B (1:800, 12216-1-AP, Proteintech), Lamp2b (1:1000, H4B4 clone, Abcam, ab25631), CD63 (1:1000, BD Pharmingen, #556019), CD9 (1:1000, BD Pharmingen, #555370), CD81 (1:1000, BD Pharmingen, #555675), Alix (1:1000, Santa Cruz, #sc-271975), TSG101 (1:1000, Novus Bio, #NB200-112). Secondary horseradish peroxidase conjugated antibodies (anti-mouse/rabbit IgG HRP-linked whole antibody donkey, GE Healthcare) were used and immunoreactive bands were visualized by using the Enhanced Chemiluminescence (ECL) (Merck Millipore). Bands of interest were quantified by using ImageJ v1.48 software (http://imagej.nih.gov).

### Immunofluorescence analysis

Cells were seeded (up to 60% confluence) on a 96 multiwell plate and incubated at 37° C with 5% CO2 in a cell culture complete medium containing 10% FBS, and 1% antibiotics. 24 hours later, fluorescent images for transgene expression evaluation were taken by using Axio Vision Imaging Software (Axiovision Rel 4.8®) on an Axio Imager M2 microscope (Carl Zeiss, Oberkochen, Germany).

### Live-cell imaging analysis

5637 bladder cancer cells were seeded (up to 60% confluence, 20,000 cells/well) in a 96 multiwell plate and incubated at 37° C with 5% CO_2_. After complete adhesion, cells were treated with 20 ug/well of CD9/CD81-RFP CS/NACS HEK293-derived EVs and incubated for 24 hours at 37 °C into a IncuCyte® S3 Live-Cell Analysis System (Essen BioScience) for real time images acquisition. Both fluorescent and phase-contrast images were automatically collected every hour respectively. Data from 3 or 9 fields per well were analyzed by IncuCyte® Software for fluorescence intensity signal and cells proliferation.

In the subsequent experiment, CD9/CD81-RFP CS/NACS HEK293-derived EVs were incubated with the green lipophilic tracer Vybrant^TM^ DiO (Molecular Probes, Eugene, OR, USA) at 37° C for 30 min (protected from light) in stirring conditions. Unincorporated dye was removed by using Exosome spin columns MW 3000 (Invitrogen). PBS containing dye only was used as negative control in cell uptake experiments. EVs were co-incubated with 5637 cells for 3 hours, both red and green fluorescence signals were acquired independently every 10 minutes.

### Confocal microscopy analysis

5637 bladder cancer cells were seeded in sub confluent conditions (130,000 cells/well) in a 24 multiwell plate and grown overnight on glass coverslips. After 24 hours, adherent cells were co-incubated with 25 ug of HEK293-derived CD9/CD81-RFP CS/NACS expressing EVs for 2 hours at 37 °C, then washed with PBS and fixed with 4% paraformaldehyde for 30 min. After extensive PBS washing to remove the residual paraformaldehyde, coverslips were mounted on microscope slides with VECTASHIELD Antifade Mounting Medium with DAPI (Vector Laboratories, Burlingame, CA, USA) and fluorescent images were acquired by using GE healthcare DeltaVision™ Ultra. Deconvolution was applied to acquired images.

### Flow cytometry analysis

Stably expressing Lamp2b-RFP CS/NACS 5637 bladder cancer cells were plated in a 10 mm dish and incubated in cell culture medium (DMEM) containing 10% FBS and antibiotics for 24 h at 37 °C under 5% CO_2_. After complete adhesion, cells were detached with 0.01% trypsin at 37 °C for 10 min, collected, and washed with PBS containing 1% FCS. Cells were then resuspended in 100 μL of PBS containing 1% FCS and acquired by AccuriTM flow cytometer (BD Biosciences). Analysis was done on 20.000 gated events per sample.

## Results

### Tetraspanins-based, RFP-fusion proteins are massively expressed by cells and sorted to EVs

**Figure 1.**
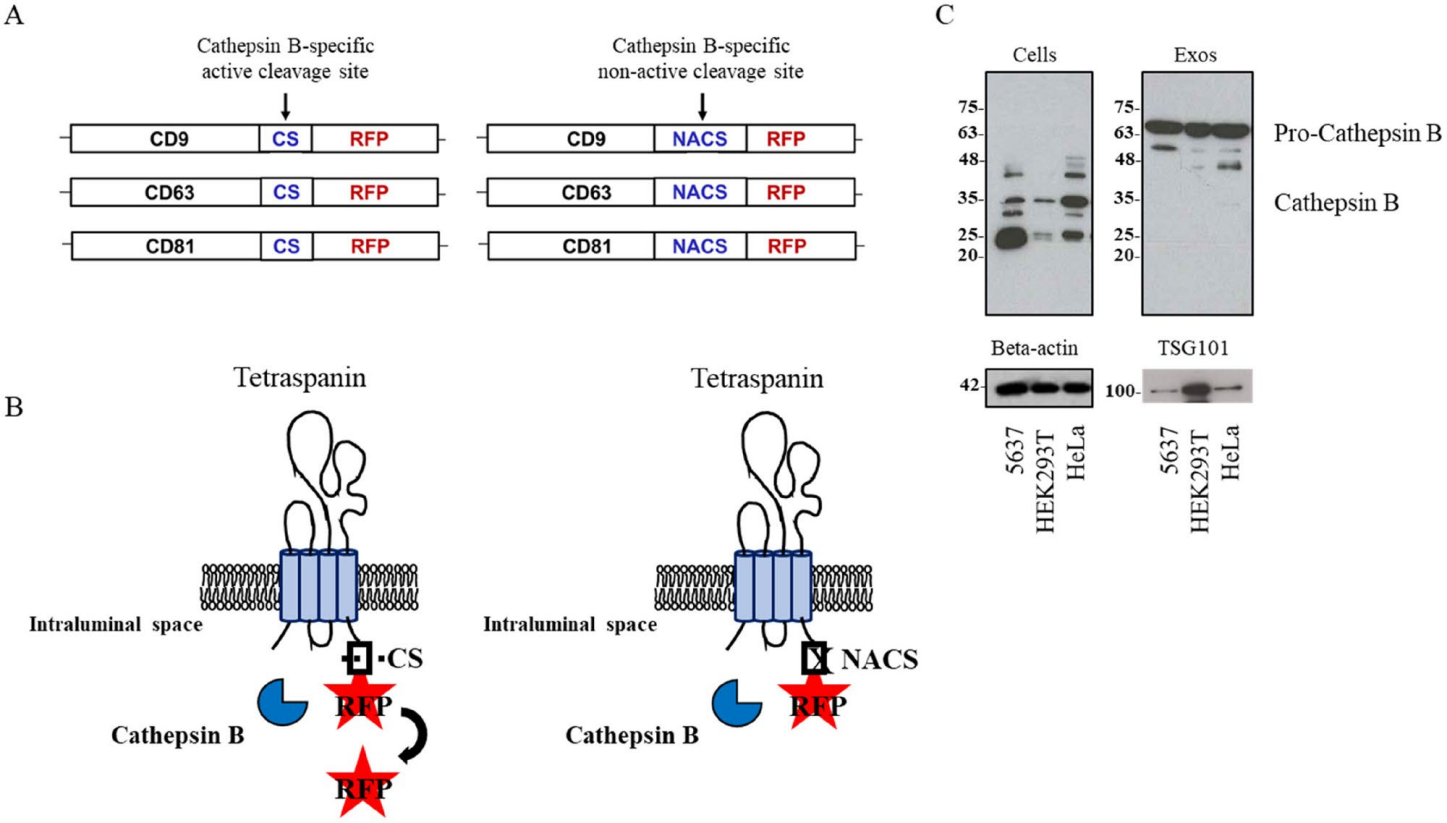
(A) Schematic representation of the tetraspanin-based recombinant proteins composition. CD9, CD63 and CD81 tetraspanins are fused to RFP reporter protein, carrying on its 5’terminus a cathepsin B-specific active cleavage site (CS) or non-active cleavage site (NACS). (B) Schematic representation showing the expected tetraspanin-based recombinant proteins orientation on EV membrane and cathepsin B-dependent RFP release. (C) Western blot analysis of cathepsin B from 5637, HEK293 and HeLa cells and respective EVs, representative of three independent experiments; detection of beta-actin for cell extracts and TSG101 for EV extracts was used as loading controls.

Aiming at the evaluation of their potential as ‘cargo-bearer’ EV markers, we exploited DNA recombinant technology to genetically engineer CD9, CD63 and CD81 tetraspanins to carry an RFP reporter protein at their C-terminus (Figure 1(A) and 1(B)). The use of a trackable payload such as RFP, in fact, allows us to, on one hand, mimic the steric hindrance of a selected cargo and, on the other hand, to follow the resulting recombinant protein fate in its entire path. In this EV-based delivery system prospect, we furthermore included a cleavage sequence for a tumor-specific cellular protease, which guarantees the selective release of the exogenous RFP form the membrane-anchored tetraspanins seizure. To this purpose, we opted for a 4 amino acidic-cathepsin B-targeting site (GFLG), which we will refer to as CS for all the rest of this study, whose coding sequence was inserted in between the CD9/CD63/CD81 and RFP. In parallel, a non-active cleavage site GGSG (NACS) mutant, which is not recognized by the protease, was prepared and used as Cathepsin B-specificity control. Cathepsin B is a cysteine protease whose massive expression has been reported in several invasive and metastatic cancers (Aggarwal and Sloane, 2014). In this work, its differential presence in cells was evidenced by western blot analysis and demonstrated to be higher in 5637 bladder and HeLa cervical cancer model cells compared to HEK293 cells, especially in its mature active form (25–35 kDa). Conversely, the EV fraction derived from the three cell lines did not show the presence of cathepsin B neither in its non-active (procathepsin B, 35–40 kDa) nor in its active form (Figure 1(C)).

**Figure 2.**
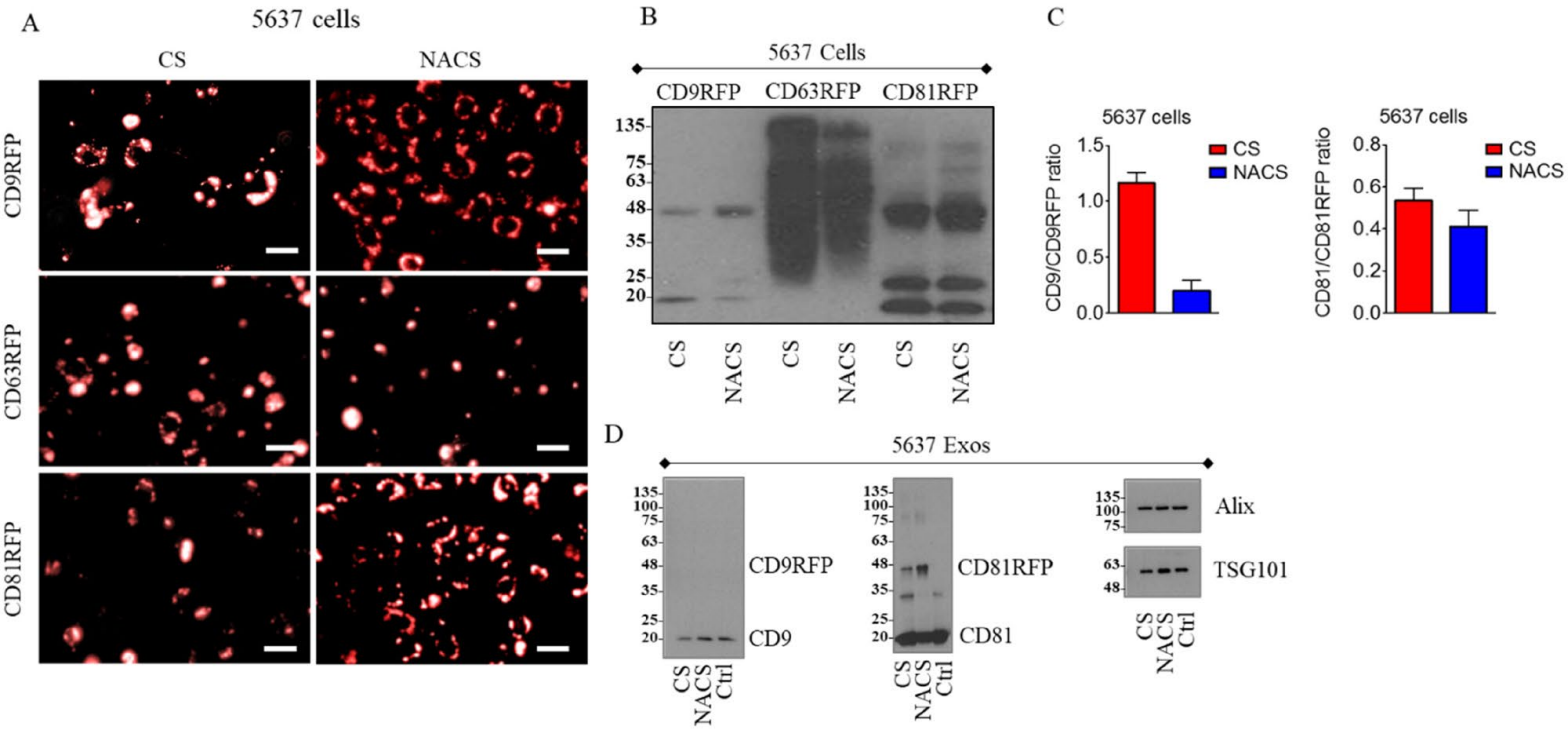
Exogenous tetraspanin-based RFP CS/NACS recombinant proteins expression levels in 5637 cells lysates and EV fraction. A fluorescence microscopy images of 5637 cells stably expressing CD9/CD63/CD81-RFP CS/NACS recombinant proteins (image magnification 10X). Representative western blot analysis comparison between CD9, CD63 or CD81 expression levels form 5637 CD9/CD63/CD81-RFP CS/NACS cells lysates (B) and EV fraction (D); Alix and TSG101 detection was used for quantitative protein normalization. C Graphs represent the relative ratios of CD9 RFP CS/NACS to basal CD9 signal as determined by densitometry. ***: *p* < 0.001 by two-tailed t-test; ns: not significant.

In order to evaluate the feasibility of our tetraspanin-based cathepsin B-targeting strategy, as a first step we expressed CD9/CD63/CD81-RFP CS/NACS recombinant constructs in 5637 cancer cell line, characterized by high cathepsin B expression level, and compared the 3 resulting phenotypes in terms of fluorescence pattern, amount of protein produced with respect to basal, and amount of protein sorted into the EV fraction (Figure 2).

We first noticed that a different fluorescence pattern and intensity could be observed in cells expressing CD9 or CD81-RFP carrying the active (CS) or non-active (NACS) cleavage site, as a more intense signal was evident in cells transfected with the NACS-RFP carrying tetraspanins compared to RFP CS-fused tetraspanins (Figure 2(A)). This difference could also be well appreciated when 5637 cells lysates were analyzed by western blot: the quantitative ratio between CD9-RFP CS or CD81-RFP CS and their respective CD9 or CD81 basal forms resulted, in fact, to be visibly lower compared to CD9-RFP NACS or CD81 RFP NACS and CD9 or CD81 basal forms, indicating a high protease specificity against the inserted active cleavage site in the bladder cancer cells expressing cathepsin B (Figure 2(B) and 2(C)). However, 5637 cells appeared to be completely inefficient in transferring both CD9-RFP CS/NACS exogenous proteins to EVs; whereas CD81-RFP CS/NACS were conveyed into EVs at a lower extent compared to CD81 alone (Figure 2(D)). On the other hand, although CD63 is one of the most used tetraspanin for EV tagging, we found that in our system its expression was often associated to round, floating cells, suggesting a major toxicity of this construct with respect to CD9 and CD81 ones. Furthermore, the presence or the absence of the cathepsin cleavage site did not affect the expression of the fusion protein in terms of fluorescence intensity or signal diffusion pattern (Figure 2(A)). The use of CD63 was even more discouraged by its appearance in western blot analysis, where the lack of a clear and well-defined band impairs the clear recognition of the recombinant product (Figure 2(B)).

In parallel, we followed a similar approach to evaluate the potential of Lamp2b as ‘cargo-bearer’ and compare it to tetraspanins. We cloned the RFP reporter gene downstream the Lamp2b coding sequence to maintain intact the signal peptide at the N-terminus, as essential for the correct sorting of the protein into the MVB compartment (Alvarez-Erviti et al., 2011), and allowing RFP orientation toward the intra-vesicle lumen. The CS/NACS was inserted between the two proteins (Figure 1(A) and 1(B), Supplementary material). 5637 bladder cancer cells transfected with the constructs showed a fluorescence signal mostly confined to round and floating cells, probably corresponding to the dead cells fraction (Figure 1(C), Supplementary material). Thus, we sought to determine if the expression levels and, consequently, the EV enrichment with the exogenous fusion proteins could be increased by the production of stably expressing cell clones. Conversely to our expectations, the antibiotic selection resulted in a complete loss of Lamp2bRFP both CS/NACS (Figure 1(D), Supplementary material) and no signal corresponding to the molecular weight of the recombinant protein could be detected in the EV fraction (Figure 1(E), Supplementary material). With the aim to exclude a possible cell type-specific degradation of the Lamp2b constructs, the same experiments were performed on HEK293 cells (Figure 1(F), Supplementary material). Still, even though a faint band corresponding to the wild type form of Lamp2b could be revealed in the EV fraction, no Lamp2bRFP signal was detectable. In addition, the inhibition of lysosomal degradation did not result in the recovery of the fusion proteins (data not shown). So, we gave up to further developing a Lamp2b-based recombinant proteins and carried on with tetraspanins-RFP fusions.

**Figure 3.**
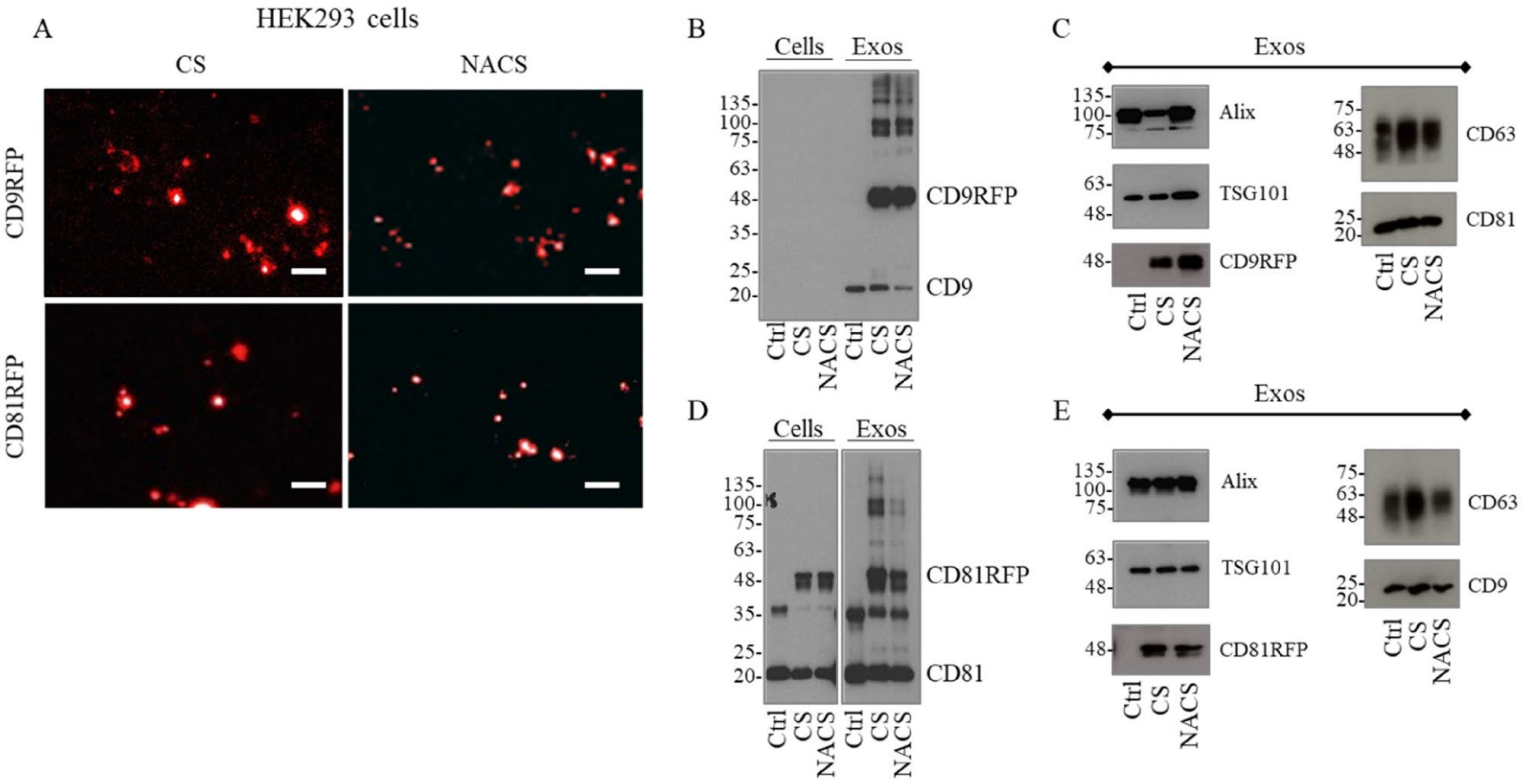
Exogenous CD9/CD81-RFP CS/NACS protein expression in HEK293 cell lysates and EV fraction. A Fluorescence microscopy images of HEK293 cells stably expressing CD9/CD81-RFP CS/NACS recombinant proteins. Representative western blot analysis comparison between CD9 (B) or CD81 (D) from CD9/CD81-RFP CS/NACS HEK293 cells lysates versus EVs. C, Representative western blot analysis comparison of CD81 and CD63 from CD9-RFP CS/NACS HEK293 derived EVs (C) versus CD9 and CD63 from CD81-RFP CS/NACS HEK293 derived EVs (E). The CD9/CD81-RFP fusion protein was detected also with anti-RFP antibody (CD9-RFP in C e CD81-RFP in E). Alix and TSG101 analysis was used for quantitative protein normalization and RFP detection to confirm the presence of recombinant proteins.

Intrigued by the observation that a cathepsin B-dependent cleavage activity on CD9-RFP recombinant protein is evident in a cathepsin B-overexpressing cancer cell line, we wondered whether the same phenotype could also be reproduced in a non-cancer cell line like HEK293 cells. Although CD9-RFP fluorescence signal in transfected cells seemed to be more prominent for NACS respect to the CS counterpart (Figure 3(A)), both exogenous CD9-RFP CS and NACS was mostly conveyed to EVs, as shown by western blot analysis (Figure 3(B)).

Indeed, a high enrichment of both endogenous CD9 and recombinant CD9-RFP CS/NACS fusions, latter corresponding to a comparable amount of exogenous RFP (Figure 3(C)), is evident in the EV fraction, while none of each could be detected in corresponding HEK293 cell lysates in these conditions (at equivalent protein amounts loaded). We could observe different enrichment efficiency for the CD81-RFP CS/NACS whose ratios to a CD81 basal level were comparable in both EVs and cell lysates (Figure 3(D) and 3(E)). In contrast to what observed within 5637 cells, both endogenous and recombinant CD81 proteins and RFP were efficiently sorted into EVs, without interference with the sorting of other tetraspanins onto the vesicles (Figure 3(C) and 3(F)). The choice of CD9 and CD81, rather than of CD63, as tetraspanins able to efficiently drive RFP fusion proteins to EVs in HEK cells was further endorsed by the published reports and in-house observation that in these cells CD63+ vesicles constitute only a minor fraction of overall released nanosized vesicles (Cashikar and Hanson, 2019; Lee et al., 2019; Fordjour et al., 2022). To further confirm the existence of cell type-dependent differences in a mechanism by which tetraspanin-based RFP-fused products are sorted into EVs, we took advantage of HeLa cells as a supplementary tumor cell model. We found that these cells produce very low amounts of both CD81 and CD9-RFP fusion proteins. A slight RFP signal could be detected only in cells expressing CD81-RFP NACS recombinant protein, whereas CD81-RFP CS signal was not detectable at all in the same experimental conditions (Figure 2(A), Supplementary material). Accordingly, by western blot analysis it was possible to observe some bands with a molecular weight falling in between 25 and 35 kDa in the total cell lysate, likely representing degradation products as they are absent in the non-transfected control (Figure 2, Supplementary material). Our analysis also evidenced the lack of conveyance of either CD9 or CD81 cargo carrying protein to the vesicular fraction (Figure 2(B) and 2(C), Supplementary material). These results further confirm that EV markers are subjected to different sorting mechanisms in healthy versus cancer cell lines, as well as between different cancer cell lines, leading to cell-specific intra- or extracellular fate.

### Tetraspanins-based, RFP fusion proteins are transferred through EVs to cancer cells

In the light of data and results collected, we next investigated if EVs deriving from CD9-RFP transfected HEK293 cells were able to horizontally transfer the exogenous cargo protein to recipient cancer cells. To achieve this goal, HEK293-derived CD9-RFP CS and NACS armed EVs were collected and their molecular and structural characterization was performed according to the guidelines of the International Society for Extracellular Vesicles (Théry et al., 2018). TEM analysis revealed the presence of round-shaped vesicles, often with a thin and even more electron-dense membrane. CD9/CD81-RFP CS and NACS vesicles displayed similar shape, electron density and size ranging from 50 to 150 nm (Figure 3(A), Supplementary material).

**Figure 4.**
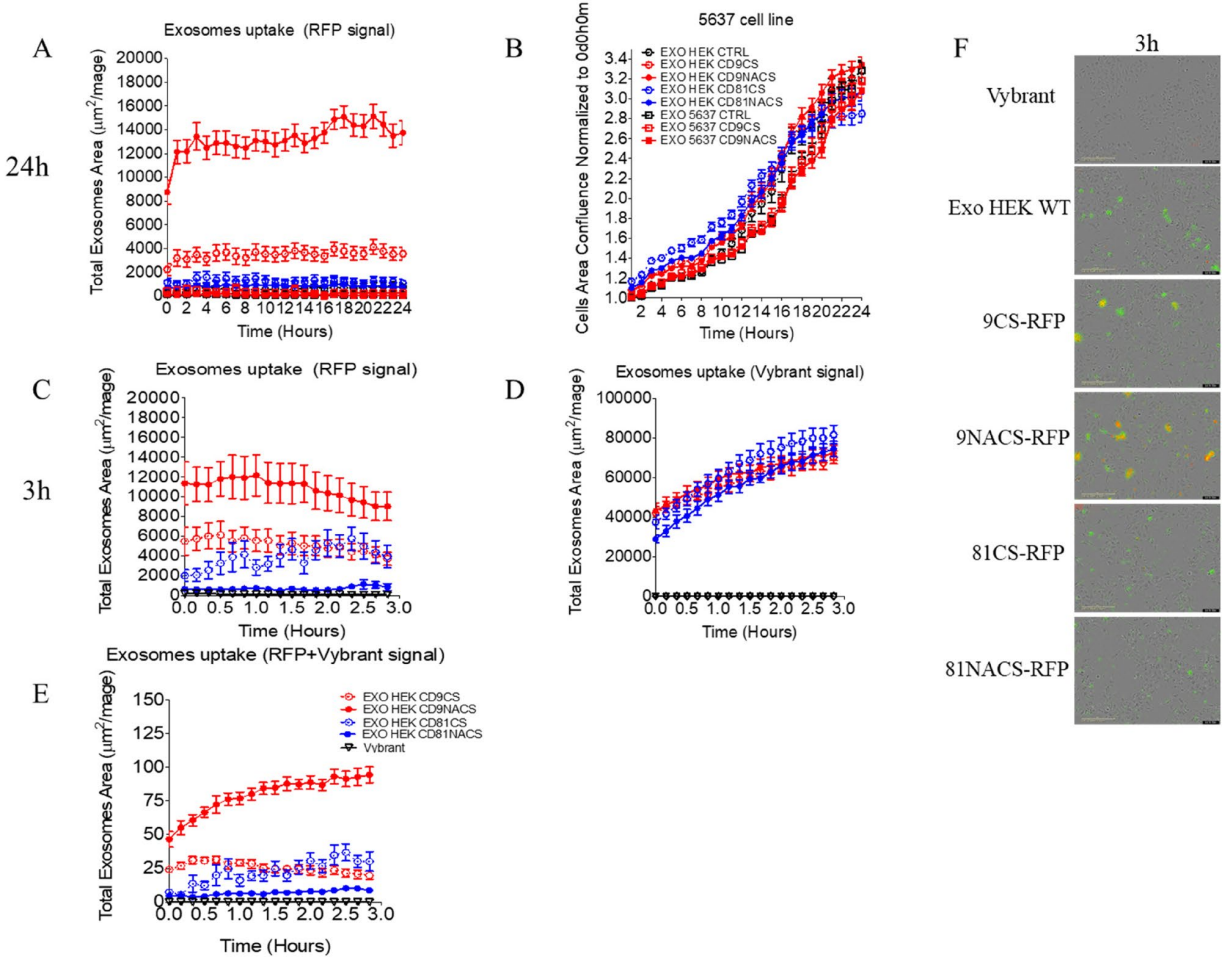
*In vitro* uptake over time of HEK293-derived CD9/CD81-RFP CS/NACS-expressing EVs by 5637 cancer cells. Panels A and B: HEK293-derived CD9/CD81-RFP CS/NACS-expressing EVs were co-incubated with 5637 cells for 24 hours into an IncuCyte imaging system; EV-derived red fluorescence signal and cell phase-contrast images were acquired once per hour and reported as total EVs area (A) and total cell area (B). Mean fluorescence signal has been obtained from six fields per well (*n* = 2). Panels C, D and E: HEK293-derived CD9/CD81-RFP CS/NACS-expressing EVs were stained with green Vybrant DiO lipophilic dye and co-incubated with 5637 cells for 3 hours into an IncuCyte imaging system; red and green EVs-derived fluorescence signals were acquired once every 30 minutes and reported as total EVs area for red (C), green (D) or color merge (E). Mean fluorescence signal has been obtained from three fields per well (*n* = 2). Panel F: Representative fluorescence images from IncuCyte analysis with merged green/red signals of EVs uptake 3 hours after incubation with 5637 cells.

NTA analysis confirmed similar number of particles/ml and vesicle size profiles among the four samples, suggesting that the EV main features are not affected by the choice of tetraspanin used in the fusion products as well as by the introduction of the cathepsin D cleavage site (Figure 3B and 3C, Supplementary material). After 5637 bladder cancer cells were co-incubated with HEK293-derived CD9-RFP CS/NACS-carrying EVs, vesicular uptake was first recorded every hour for 24 hours as red fluorescence emission. Any possible autofluorescence background was excluded by using EVs from WT HEK293 as a control. As shown in Figure 4(A), 5637 cells were able to capture HEK293-derived CD9-RFP CS/NACS carrying EVs already at the earliest time points after administration, displaying an enhanced fluorescence signal which was higher compared to 5637 receiving HEK293-derived CD81-RFP CS/NACS (Figure 4(A)).

**Figure 5.**
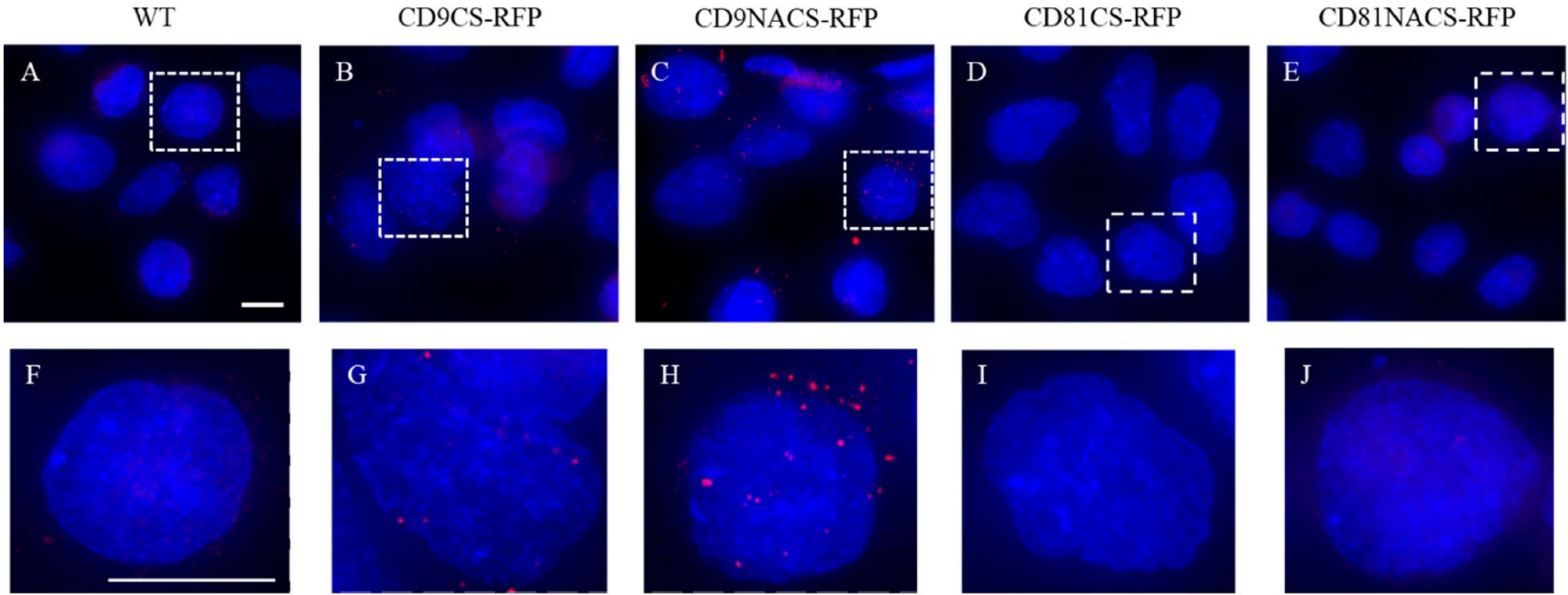
*In vitro* uptake and horizontal transfer of CD9/CD81-RFP CS/NACS cargo from HEK293-derived EVs to 5637 recipient cells. A Representative fluorescence images form confocal microscopy analysis of 5637 cells incubated for 24 hours with HEK293-derived CD9/CD81-RFP CS/NACS expressing EVs (A–E). DAPI. Scale bar 20 µm. Images are representative of three independent experiments. Figures F–J are the full view of the cells imaged in the inset A–E.

The fluorescence signal of both CD9 constructs was maintained over time, with CD9-RFP CS treated cells showing a lower signal with respect to the NACS counterpart, that, in turn, remained higher compared to CD81 fusions. On the contrary, no signal was detected in cells supplemented with 5637-derived EVs (data not shown). Importantly, 5637 cell proliferation rate was not altered by the treatments (Figure 4(B)). In order to further clarify the uptake timing and better elucidate the mechanism by which RFP transfer to recipient cells is increased when shuttled by CD9 NACS, HEK293-derived WT or CD9/CD81-RFP CS/NACS EVs were labeled with a green lipophilic tracer (Vybrant DiO) before administration to cells. The intensity of red fluorescence derived from RFP and the green one from Vybrant DiO were monitored and acquired in live imaging every 10 minutes for 3 hours and compared (Figure 4(C), 4(D) and 4(E)). In accordance with our hypothesis, the EVs uptake rate and timing were similar in every condition used, as confirmed by the green Vybrant signal detected (Figure 4(D)). RFP signal from CD9 NACS-carrying EVs was likewise enhanced and superimposable with the Vybrant one, while CD9-RFP CS carrying EVs resulted in a less intense red punctuation in recipient cells (Figure 4(E) and 4(F)). Finally, the vesicles uptake and internalization by 5637 cells were further confirmed by confocal microscopy, which also highlighted a major fluorescence signal detection in the case of CD9-RFP carrying EVs compared to the CD81-RFP containing EVs, with the CD9-RFP NACS carrying EVs administration resulting in higher amount of red dots per cell, confirming what was observed in live imaging (Figure 5).

The overall data indicate that the CD9-RFP is the best recombinant protein to be expressed in HEK293 cells, as it is massively sorted onto EVs and correctly transferred to recipient cells where the destiny of an RFP tag remains sensitive to cell specific enzymatic activity, paving thus the way to the EVs-based delivery and selective release of therapeutic proteins in cancer cells.

## Discussion

One of the major advantages of using cell derived vesicles rather than chemically synthetized liposomes concerns the possibility to completely evade the immune system response and leverage nature-perfectioned routes to vectorize molecules between cells (La-Beck and Gabizon, 2017). In fact, patient-derived healthy cells can be isolated and cultured *in vitro* with the purpose to both genetically engineer them to produce therapeutic EVs or induce the production of EVs that can be manipulated *ex vivo*. This approach leads to the prospect of personalized and well tolerated treatments, as the drug nanocarriers would be recognized as self-molecules by the patient body, achieving an improved circulation time and bioavailability. The *ex vivo* electroporation is currently the most used process of cargo loading into extracellular vesicles. However, this system has been demonstrated to affect the quality of the final product, including inducing alterations of the structural properties and/or of the chemical characteristics of the compound (Walker et al., 2019).

**Figure 6.**
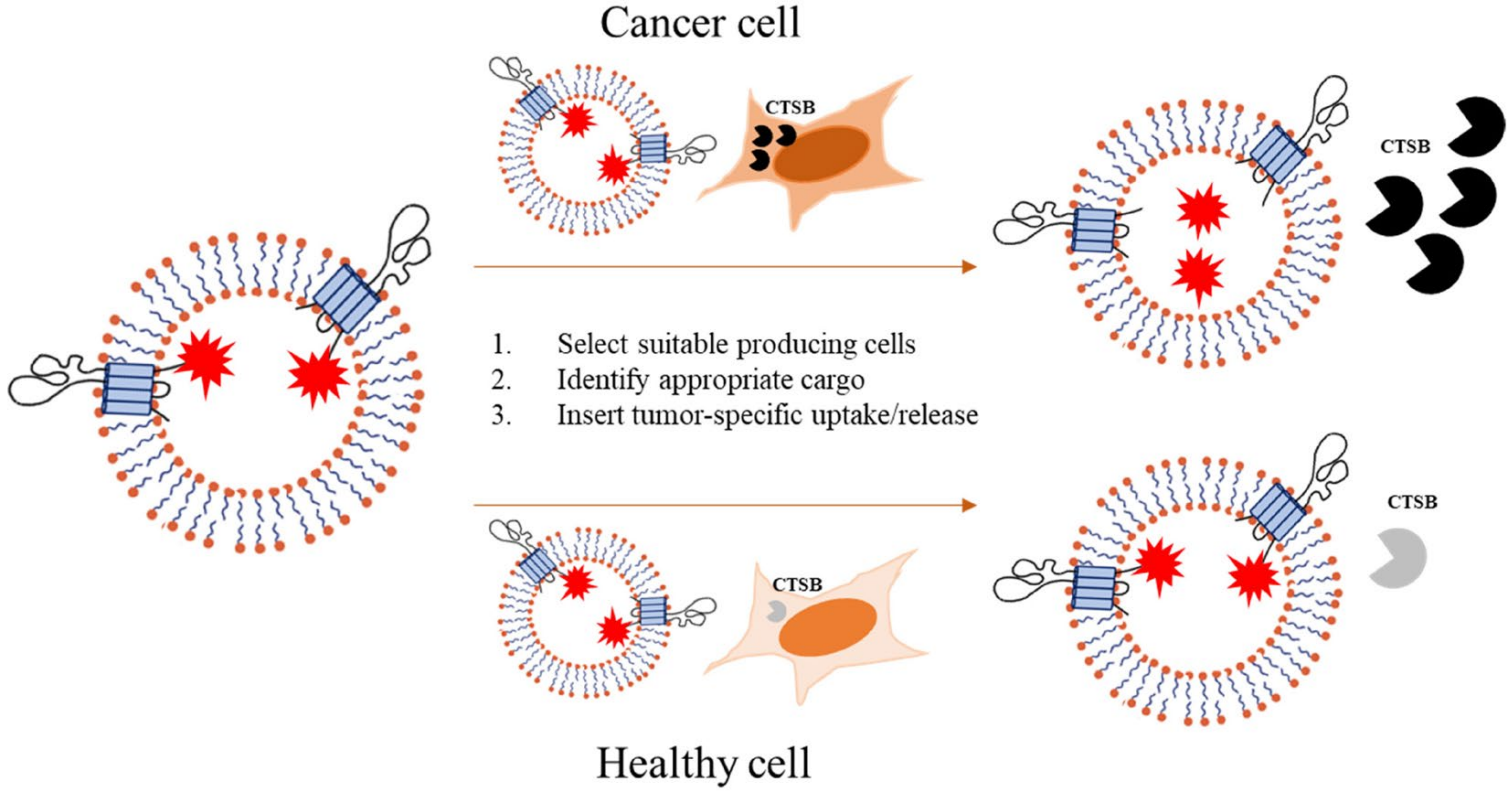
Model of engineered EVs carrying a tetraspanins-fused cargo, whose release into recipient cells is dependent on cathepsin B expression. CTSB: cathepsin B.

In this work, we genetically engineered cells with the aim to produce therapeutic competent EVs. In particular, as a proof of concept, we took advantage of an RFP reporter protein as a cargo, in order to both trace EVs and recapitulate the expression of a hypothetical protein-based drug. We firstly evaluated the efficiency of different EV transmembrane proteins as potential ‘payload bearer’ molecules. In particular, the ideal one should not suffer from the presence of a ‘bulky cargo’, that means that it should be correctly folded and sorted to its final destination and should not be sent to the degradation pathway. So far, the different orientation throughout the plasma membrane has influenced the choice of EV markers as cargo-bearer molecules, as it is essential to set up a self-assembling therapeutic that does not need any further *in vitro* modifications. Lysosomes associated membrane protein 2b (Lamp2b), for instance, is a membrane protein with a single transmembrane domain, already shown to be efficient in displaying targeting moieties on the surface of EVs (Alvarez-Erviti et al., 2011; Tian et al., 2014; Wang et al., 2017). As aforementioned, the modification of the N-terminus domain of the protein by DNA recombinant technology leads to the addition of a functional peptide that improves recombinant protein-carrying vesicles specific uptake by target (including cancer) cells. Conversely, the modification of the C-terminus of Lamp2b, might enable the expression of a protein cargo facing the lumen of the vesicle, making it, therefore, protected from degradation. Tetraspanins, in turn, have been modified on their C-terminus in order to carry a fluorescent reporter protein and commonly utilized in laboratory methods to trace the EVs fate. To warrant the selective release of the payload into recipient cells, we designed a smart-prodrug system, taking advantage of a six amino acids sequence (inserted in between the C-terminus of the transmembrane protein and an RFP reporter) containing a dipeptide specific for the protease activity of cathepsin B (Dubowchik and Firestone, 1998). Cathepsin B is known to be massively expressed in cancer cells, but not in healthy cells, being involved in many steps of cancer progression (Aggarwal and Sloane, 2014). In cancer, cathepsin B is found in lysosomes as well as in vesicles, throughout the cytoplasm and at the cell periphery (Gondi and Rao, 2013; Ruan et al., 2015). Smart prodrugs systems based on doxorubicin and other small molecules, targeting the protease activity of cathepsin B in tumor microenvironment have been demonstrated to be highly efficient in killing metastatic cancer cells while sparing normal cell (Dubowchik et al., 2002; Dubowchik et al., 1998; Zhong et al., 2013). The goal of these studies was to selectively release the drugs in the tumor microenvironment, exploiting the secreted cathepsin B form, which associates to the plasminogen cascade, the latter leading to the activation of factors involved in the extracellular matrix degradation and cancer cells invasion. Herein, we proposed a similar strategy directed at allowing the EV cargo protein specific release in the receiving cancer (but not healthy) cells with a high cathepsin B expression (Figure 6). Moreover, such a release of a cargo protein in its free form within the recipient cell would surpass the criticism to a conventional recombinant EV mediated delivery approach that would require the cargo molecule to be irreversibly anchored to the membrane, therefore likely limiting its range and functionality *in situ*.

Next, we compared different potential donor cells, either healthy or cancerous, for both the expression of such exogenous molecules and their sorting into EVs. The main aspect to consider is that cells producing therapeutic EVs should be able to produce a conspicuous number of extracellular vesicles. Therefore, as a proof of principles, we selected 5637 (bladder cancer), HeLa (cervical cancer) and the HEK293 (non-immortalized, embryonal kidney healthy cell line), all prior acknowledged for their high transfection efficiency and their capacity to produce a high amount of EVs. 5637 and HeLa tumor cell lines were found to be positive either for the active cleaved and the zymogen forms of cathepsin B, while EVs deriving from the same cell lines were negative for the enzyme. HEK293 are endowed with a highly reduced amount of cellular cathepsin B, as expected. Additional appeal of the healthy producer cells for the therapeutic EVs would be the lack of immunogenic or tumorigenic components. Beyond being a popular research model also in EV field, HEK293 is acknowledged and safe source for production of commercially deployed recombinant proteins and viral vectors. These cells are also appealing and sustainable for translation and scale up due to the open access (they are not proprietary) and large cell banks available. When 5637 and HEK293 cells were transfected to produce the Lamp2b-based recombinant protein of interest, both lines underwent a sudden drop of the signal as soon as they were exposed to an antibiotic clone selection and the inhibition of lysosomal degradation did not allow recovering the fusion proteins. Therefore, this phenomenon could rely on a cell adaptation to the possible cytotoxicity of the constructs, which was not related to the presence of the cleavable sequence susceptible to cathepsin B protease activity. In fact, the same loss of signal was evident in both cells transfected with the construct carrying the active CS and its non-active mutant. Consequently, in order to verify such a hypothesis, we cloned the RFP gene carrying the CS or NACS sequences downstream to tetraspanins. A high expression of these recombinant proteins could be achieved in both 5637 and HEK 293 cells. Despite overall similar recombinant proteins levels detected in cells transfected with CS or NACS containing constructs, different fluorescence pattern was observed in tumor cells, indicating the functionality of the cathepsin sensitive site. Indeed, in cells expressing NACS fusions the bright masses are visible, corresponding to intracellular membranes anchored RFP, while signal observed in CS containing RFP fusions is lower likely due to the fact that RFP is cleaved and diffuses from the membranes. In addition, the constructs were not only delivered to the extracellular vesicles fraction, but also correctly processed by cathepsin B protease as demonstrated by the differential ratio between the fusion products carrying the CS or NACS sequences in cancer cells. Importantly, we could speculate the existence of different intracellular sorting pathways of recombinant proteins in the cell types we used. HEK293 cells were able to effectively direct CD9 fused RFP to the extracellular vesicles fraction, while CD81 fused RFP constructs were less efficiently sorted into EVs. Further investigations are needed to define in depth the mechanisms of fusion protein sorting to EVs. 5637 cells processed both the CD9 and CD81 constructs carrying the cathepsin B specific cleavage site, but did not (or minimally did) sort them into the EVs. Remarkably, only the CD9 fused RFP was correctly managed by 5637 cancer cells, whereas transfection with CD81 variant resulted in a series of possible degradation products. This result indicates that 5637 cells represent a good model of cancer recipient cell to test the activity of cathepsin B-specific smart prodrugs, showing a highly specific intracellular activity of the enzyme. On the other hand, HEK293 cells are a suitable system for the production of EVs carrying the cathepsin B smart prodrug, as the enzymatic activity is not detectable at a cellular level and the exogenous proteins are correctly delivered to the EV fraction. Based on these evidences, we coupled the successful activity of 5637 cytosolic cathepsin B demonstrated on CD9/CD81-RFP fusions to the HEK293-derived CD9/CD81-RFP bearing EVs, in order to evaluate a potential horizontal cargo transfer. CD9/CD81-RFP bearing HEK293-derived EVs is efficiently captured by 5637 recipient cells after short time points. The staining of vesicles membranes with green tracer and detection of merged green and red signals in recipient cells, confirms that the transfer of recombinant RFP proteins is mediated by the uptake of intact EVs. Uptake of CD9-RFP bearing EVs resulted in a more intense signal with respect to that from CD81-RFP fusions, confirming an impaired sorting and partial degradation of the latter in recipient tumor cells. In addition, less intense punctuated fluorescence signal was observed upon the uptake of CD9-RFP CS carrying EVs with respect to their non-cleavable counterpart, confirming not only the internalization, but also the tumor specific cell-processing of the EV cargo. Indeed, since we demonstrated that a comparable amount of the two exogenous proteins is transferred to the EV fraction, and the EV uptake rate was equal and steady for both fusions containing vesicles, the reduced fluorescence signal intensity could be due to the high cellular levels of cathepsin B, which, once recognized the CS sequence, induces the release of the most of the RFP cargo from the membrane and its spread inside the cell. It is known that the quantifiable discrimination of diffused cytoplasmatic fluorescent proteins is challenging at low concentrations (in a micromolar range) while the same low expression levels are instead detectable if RFP is targeted to discrete subcellular compartments such as membranous organelles (Niswender et al., 1995).

Finally, we also observed that the sorting of recombinant cargo to the vesicles does not interfere with the sorting of other membrane constituents (other tetraspanins) to the EV surface. The preserving of EV membrane architecture and surface display is important in targeting strategies, therefore the loading approach should not affect the EV intrinsic or acquired tropism.

## Conclusion

We documented the effective uptake of CD9-RFP NACS/CS bearing EVs by 5637 cancer cells as proven by the detection of red spots by confocal microscopy, indicating that the uptake kinetic is extremely rapid and the captured signal is stable as it resides inside the cells overtime. Taken together these results suggest that tetraspanins-based recombinant proteins are able to be massively expressed by HEK 293 cells and conveyed to the extracellular vesicles fraction. Cathepsin B specific smart prodrug system appeared to be a promising strategy in releasing a protein drug specifically to cancer cells when associated to the CD9 tetraspanin. Further studies will apply this approach to particular and potent drugs that can benefit from a delivery to tumor cells in a free and effective form. The current findings open new perspectives for the production of smart drugs for therapeutic purpose.

## Supplementary Material

Supplemental Material
