# Supplementary material for: The cell type dependent sorting of CD9- and CD81 to extracellular vesicles can be exploited to convey tumor sensitive cargo to target cells
Source: Drug Deliv. 2022 Dec 29;30(1):2162161. doi: 10.1080/10717544.2022.2162161 (PMC9809379; doi:10.1080/10717544.2022.2162161)
Supplement: Supplemental Material [file IDRD_A_2162161_SM3065.docx]

Supplementary materials

**The cell type dependent sorting of CD9- and CD81 can be exploited to convey tumor sensitive cargoes into extracellular vesicles.**

Stefania Zuppone^1^, Natasa Zarovni^2^, Riccardo Vago^1,3^

^1^Urological Research Institute, Division of Experimental Oncology, IRCCS San Raffaele Scientific Institute, Milano, Italy.

^2^ Exosomics S.p.A, Siena, 53100, Italy

^3^Università Vita-Salute San Raffaele, Milano, Italy


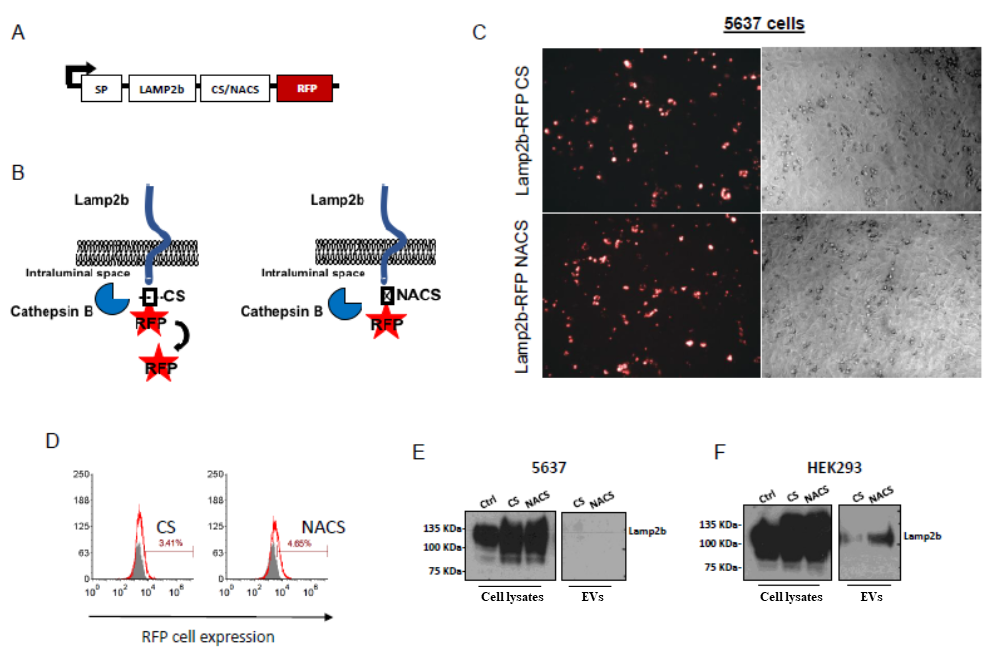


**Supplementary Fig. 1**. Exogenous Lamp2b-RFP CS/NACS recombinant proteins expression in 5637, HEK293 cells and EVs. A Schematic representation of the Lamp2b-based fusion proteins composition; Lamp2b carrying an N-terminal signal peptide (SP) is fused to RFP reporter protein, carrying on its 5’terminus a cathepsin B-specific active cleavage site (CS) or non-active cleavage site (NACS). B Schematic representation showing the expected Lamp2b-based recombinant proteins orientation on EV membrane and cathepsin B-dependent RFP release. C Representative fluorescence (left panels) and bright field (right panels) microscopy images of 5637 cells 24 hours after transient transfection with Lamp2b-RFP CS/NACS encoding plasmids. D 5637 cells stably expressing Lamp2b-based recombinant proteins (RFP CS/NACS) were analyzed by flow cytometry for Lamp2bRFP-CS/NACS expression. Lamp2bRFP-CS/NACS expressing 5637 clones selection was made by using G418 antibiotic for 1 month. E, F Representative western blot analysis of Lamp2b-RFP CS/NACS expressing levels in 5637 and HEK293 cell lysates and corresponding EV fractions.


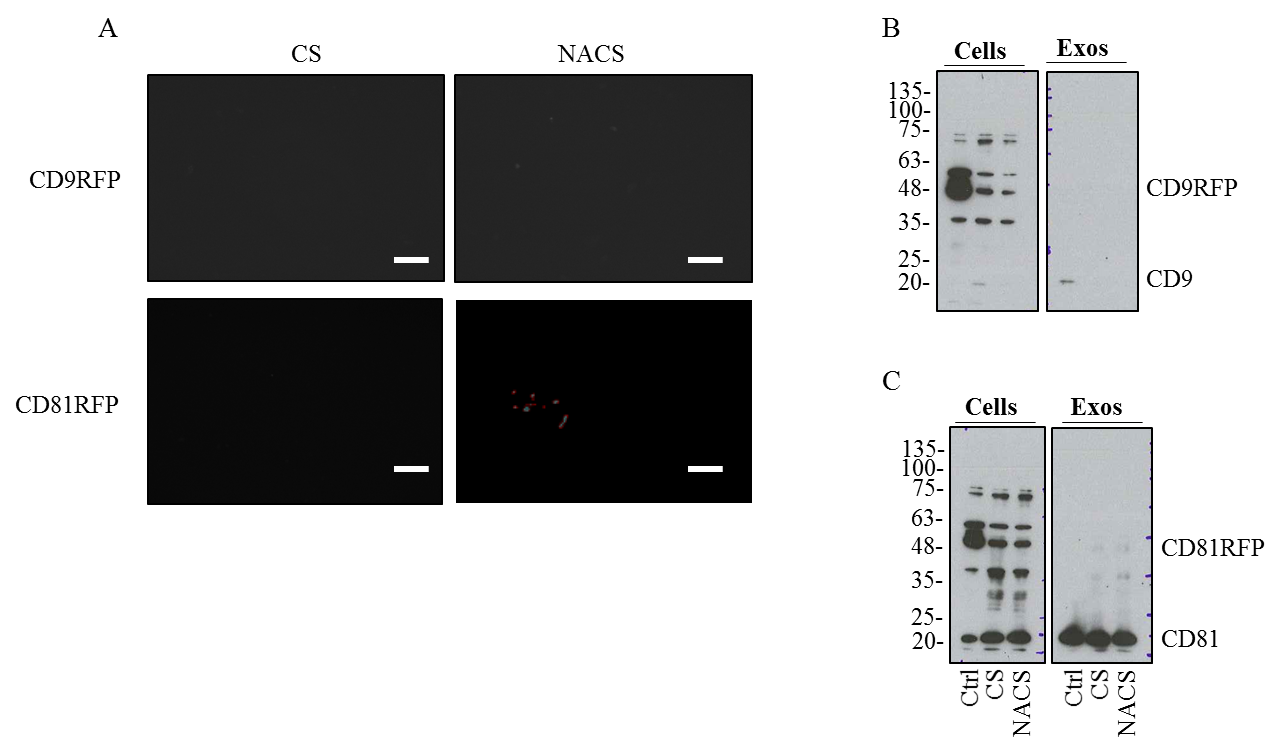


**Supplementary Fig. 2. Exogenous CD9/CD81-RFP CS/NACS protein expression in HeLa cell lysates and exosomal fraction.** A Fluorescence microscopy images of HeLa cells stably expressing CD9/CD81-RFP CS/NACS recombinant proteins. B, C Representative western blot analysis comparison between CD9 or CD81 from CD9/CD81-RFP CS/NACS HeLa cells lysates versus exosomes.


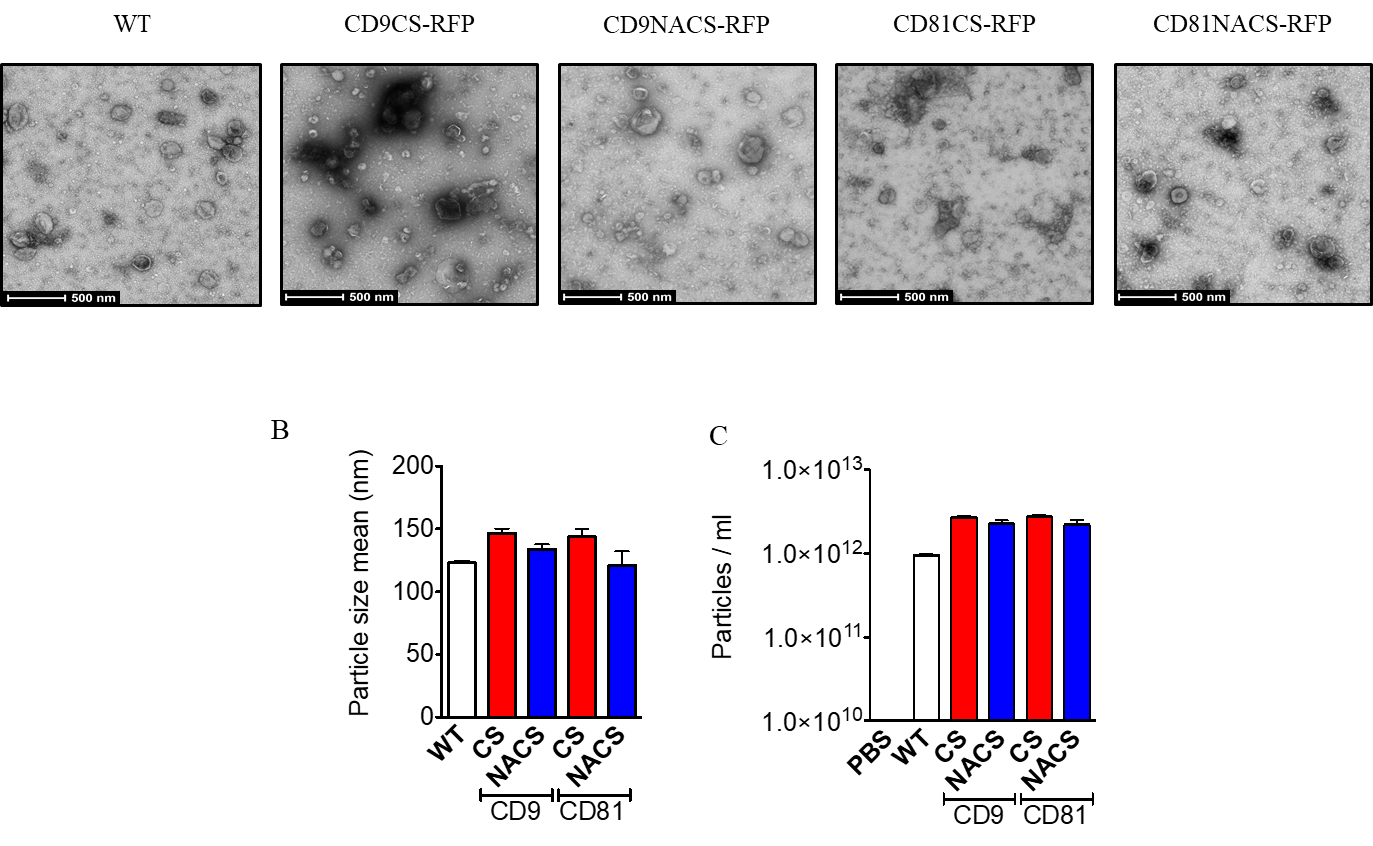


**Supplementary Fig. 3. Structural characterization of EVs derived from HEK stably expressing CD9/81 NACS/CS fusion proteins.** A Transmission electron microscopy images of EVs from CD9-CS/NACS or CD81-CS/NACS stably expressing HEK293 cells. EVs derived from HEK293 WT cells were used as control. Scale bars: 500 nm. B, C Bar graphs show the results of nanotracking analysis (NTA) of CD9/81-RFP NACS/CS HEK293 derived EVs. Concentration are defined as particle/ml (left panel) and size distribution (right panel); PBS was used as control. Results are representative of four independent experiments and shown as mean ± SEM.
